# Belonging in crisis: A mixed‐methods exploration of school disruption among marginalized girls in India

**DOI:** 10.1111/jora.70327

**Published:** 2026-11-14

**Authors:** Ananya Tiwari, Nidia Ruedas‐Gracia

**Affiliations:** ^1^ Department of Educational Psychology Texas A&M University College Station Texas USA; ^2^ Department of Educational Psychology, College of Education University of Illinois Urbana‐Champaign Illinois USA

**Keywords:** bioecological systems theory, intersectionality, marginalized girls, mixed methods research, school belonging, school closures

## Abstract

School closures can disrupt not only educational access but also the relational and institutional conditions through which adolescents experience school belonging. This sequential mixed‐methods study examined how COVID‐19–related school closures reshaped school belonging among marginalized adolescent girls in rural India. Guided by bioecological systems theory and intersectionality, we integrated surveys administered in 2021 (*N* = 90) and 2022 (*N* = 60) with two waves of focus group discussions. School belonging was conceptualized as a multidimensional, ecological, and relational experience reflected in peer and teacher connection, recognition, perceived fit, opportunities for agency, and aspirational attachment to school. Findings showed that belonging was increasingly constrained as closures continued. Girls reported growing household responsibilities, gendered restrictions on digital access, weakened contact with teachers, and heightened exposure to early marriage discussions; reported family conversations about marriage increased from 2% in 2021 to 20% in 2022. Sibling relationships, particularly girls' accounts of brothers' authority over household labor and phone access, revealed how broader gender norms were enacted within everyday family interactions. At the same time, peer relationships, teacher recognition, memories of residential school life, and educational aspirations sustained girls' connection to school despite prolonged physical absence. Girls consistently contrasted school as a space of relational connection, agency, and future possibility with home environments characterized by greater gendered constraints. The findings demonstrate that school belonging during crisis is dynamically produced across individual, relational, structural, and temporal contexts and underscore the importance of gender‐transformative approaches to educational continuity and post‐crisis recovery.

## INTRODUCTION

The COVID‐19 pandemic has disrupted adolescent development worldwide (Nearchou et al., 2020; Rousseau & Miconi, 2020), with especially severe consequences for structurally marginalized youth. In India, school closures affected over 253 million students, disproportionately impacting rural girls who face intersecting barriers related to gender, caste, class, and geography (Nandi et al., 2023). Like other minority youth globally, these girls experienced not only educational disruption but diminished access to psychosocial resources critical for well‐being and identity development (Miconi et al., 2021; Raifman & Raifman, 2020). Existing gendered norms—such as prioritization of domestic work and restricted mobility—intensified during this crisis, heightening dropout risk and early marriage (Nandi et al., 2023; Yukich et al., 2021).

School often offers girls a rare space to imagine futures beyond their ascribed roles at home (Murphy‐Graham & Lloyd, 2016). When this space was lost, their sense of school belonging—the extent to which they felt accepted, valued, included, and supported within the school community—was also undermined, with consequences for their self‐concept and emotional resilience (Allen et al., 2018; Arslan, 2018; Goodenow & Grady, 1993). Yet, few studies have examined how crisis‐related school closures reshape belonging and development for girls navigating intersecting structural inequalities.

This sequential mixed methods study investigates: *How was school belonging disrupted and sustained among marginalized adolescent girls in rural India during prolonged COVID‐19 school closures?* Guided by a bioecological and intersectional lens, we specifically examine: (1) how household labor, digital access, and emotional well‐being shaped girls' connection to school; (2) how relationships with parents, siblings, peers, and teachers supported or constrained school belonging; and (3) how gender norms, caste/class inequality, early marriage risk, and prolonged time away from school influenced girls' educational aspirations and desire to return.

Based on prior research on school belonging, gendered educational inequality, and bioecological systems theory, we expected that crisis‐related school closures would weaken girls' school belonging by increasing household responsibilities, limiting access to digital learning, heightening emotional distress, and reducing everyday contact with peers and teachers. We further expected that relational support, especially from peers, teachers, and family members, would help sustain connection to school, whereas gendered household norms, sibling gatekeeping, early marriage discussions, and prolonged time away from school would constrain belonging.

## METHODOLOGICAL CONTRIBUTION OF THE STUDY

This study presents a culturally grounded, iterative configuration of mixed methods for examining a relational construct among adolescents underrepresented in developmental science. Its methodological contribution lies in aligning construct operationalization, instrument development, qualitative follow‐up, and cross‐method integration. Because existing standardized measures did not capture the crisis‐specific ecological conditions through which marginalized girls experienced school belonging, we synthesized COVID‐19 research and coded publicly available materials containing accounts from rural adolescent girls and youth advocates to identify relevant domains. These domains informed a survey, translated into Hindi and reviewed by community experts for linguistic clarity and contextual relevance. Survey findings subsequently informed focus group discussions. The two strands were integrated across bioecological levels and two time points through narrative weaving and joint displays (Fetters et al., 2013). This design provides a transferable sequence for investigating constructs whose meanings and determinants are culturally and structurally situated.

## THE UNDERREPRESENTED REALITIES OF MARGINALIZED RURAL GIRLS IN INDIA

Despite global efforts to advance gender equity in education, rural adolescent girls in India—particularly those from religious minority communities and marginalized caste and low‐income families—remain structurally underrepresented in national education data and international scholarship (Arnett, 2008; Tiwari & Goodnight, 2024). This study focuses on girls enrolled in Kasturba Gandhi Balika Vidyalayas (KGBVs), government residential schools established in 2004 to serve the “hardest to reach” girls in Educationally Backward Blocks (NITI Aayog, 2015). Our research was conducted in the predominantly Hindi‐speaking Mewat district of Haryana, northern India.^1^ Mewat is one of India's most educationally deprived regions, with a female literacy rate of 36% (Government of India, 2011). KGBV schools here serve predominantly first‐generation learners, the majority of whom belong to Muslim communities, where strict patriarchal norms govern girls' mobility and aspirations (Roy, 2015).

Intersecting inequalities—including low parental literacy, low‐resource households and communities, precarious parental employment, religious minority status, caste‐ and class‐based marginalization, gendered labor expectations, restricted mobility, child marriage norms, geographic isolation, and the institutional marginalization of KGBV schools^2^—create severe structural barriers to educational continuity and school belonging. The COVID‐19 crisis further exacerbated these inequalities. Although scholarship has examined the pandemic's health effects on vulnerable populations (Nearchou et al., 2020), considerably less is known about the social determinants of crisis impacts for adolescent girls experiencing compounded marginalization. This study addresses that gap by documenting the experiences of girls displaced from residential schools—spaces of belonging, aspiration, safety, and identity—back into household environments where patriarchal norms often constrained their agency. Using a socio‐ecological lens and culturally responsive mixed methods, the study responds to calls to decolonize human development science (Abubakar et al., 2024; Arnett, 2008) and center perspectives from the Global South. The findings illuminate how intersecting marginalities shape school belonging and how broader systemic exclusions undermine educational justice during crises.

## COVID‐19 AND GIRLS' EDUCATION IN INDIA

COVID‐19 reversed decades of progress in girls' education, with an estimated 10 million additional adolescent girls globally at risk of child marriage due to pandemic‐related disruptions (UNICEF, 2021). In India—the country with the second‐highest number of confirmed COVID‐19 cases (over 44 million) and more than 530,000 reported deaths (World Health Organization, 2023)—the effects were especially severe. More than 320 million learners were affected by school closures, and many rural girls were pulled out of school for domestic work or early marriage (UNESCO, 2021). The crisis exacerbated poverty, gender‐based violence, and isolation, all of which undermined girls' education and heightened their vulnerability (UNESCO, UNICEF, & World Bank, 2021).

Research on crisis‐related school disruption shows that disasters can interrupt schooling through closures, infrastructure damage, displacement, and disruptions to children's routines and support systems, with consequences for academic performance, emotional well‐being, and educational continuity (Gibbs et al., 2019; Lai et al., 2016; Miller & Hui, 2022). This insight is particularly relevant in Haryana's Mewat district, where school closures unfolded within an already educationally marginalized context. Meo and Chanchal (2021) found that COVID‐19 school closures deepened inequities in access to online learning among marginalized students, largely due to limited access to digital devices, internet connectivity, technical knowledge, and household support. For KGBV girls, therefore, school closure meant not only the loss of classroom instruction but also the loss of a residential school environment that supported peer connection, structured routines, and educational continuity.

## SCHOOL BELONGING AND BIOECOLOGICAL SYSTEMS THEORY

School belonging—a feeling of relatedness and connection to a social group or system in which one feels valued or important (Hagerty et al., 1992; Hill, 2006)—is central to adolescents' well‐being, self‐efficacy, academic performance, and educational engagement (Maurizi et al., 2013; Neel & Fuligni, 2013; Rejaan et al., 2021; Uwah et al., 2008). In this study, belonging is understood not as a fixed individual feeling but as a relationally produced and structurally mediated experience shaped through interactions with peers, teachers, families, and broader sociocultural systems (Johnson, 2022, 2026; Strayhorn, 2018). This perspective is especially important in crisis contexts, where loss of physical access to school may disrupt both instruction and the relational and institutional conditions that sustain belonging.

Drawing on later developments in Bronfenbrenner's bioecological theory, we examine how development is shaped through dynamic interactions among person, process, context, and time within nested ecological systems (Bronfenbrenner & Morris, 2006; Tudge et al., 2009). Accordingly, school belonging was examined through its ecological determinants, correlates, and expressions rather than through a standardized psychometric measure. We focused on (a) individual factors, including demographic characteristics, household chores, educational resources, and emotional health; (b) micro‐level relationships with parents, siblings, peers, and teachers; and (c) macro‐ and chrono‐level conditions, including gender norms, household expectations, investment in schooling, and prolonged school closure (see Figure 1).

**FIGURE 1:**
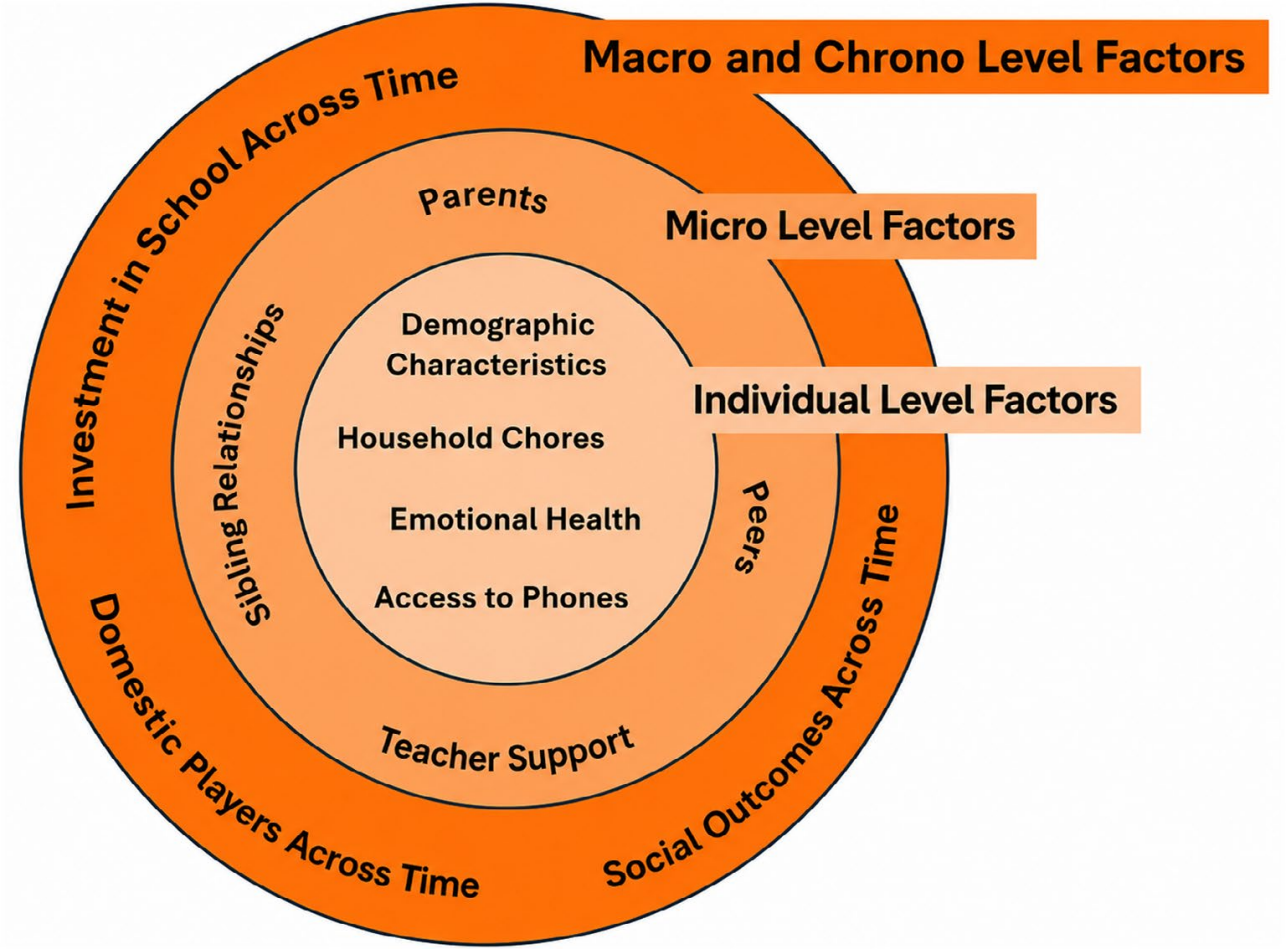
Sociocultural Environment Factors for Assessing School Belonging Adapted from Bronfenbrenner's Bioecological Model. The substance of levels is not mutually exclusive; instead, they overlap, e.g., there is an overlap between access to phones (individual) and siblings (micro; brothers' power in deciding if a girl gets phone use), which we discuss later.

These levels interact in shaping belonging. Individual characteristics influence experiences of inclusion within educational settings (Hayes et al., 2017), while supportive relationships with parents, siblings, and caregivers promote academic and emotional stability, and peer and teacher relationships strengthen engagement, trust, and connection to school (Allen et al., 2018; Longaretti, 2020; Porter et al., 2021). Macro‐ and chrono‐level influences, including cultural norms and disruptions such as COVID‐19, interact with these individual and relational processes but remain comparatively underexamined because of their methodological complexity (Allen et al., 2018; Brown Kirschman & Karazsia, 2014). Our systems‐oriented approach therefore examines how belonging emerged through interactions across ecological levels during profound sociocultural disruption.

Few studies have examined school belonging during COVID‐19. Existing research suggests that online platforms provided some students with temporary opportunities for connection and belonging, particularly in economically developed contexts (Potts, 2021; Reed & Dunn, 2021). Such work, however, often assumes continued access to online learning. This contrasts sharply with marginalized adolescent girls whose belonging was disrupted not only by physical school closures but also by sociocultural constraints that online modalities could not remedy.

## METHOD

A central aspect of our research design was cultural appropriateness. The preparatory phase (Table 1) began with a synthesis and thematic analysis of global COVID‐19 literature, followed by coding of published interviews with rural adolescent girls to develop the survey questions (see Supplementary Material A), which subsequently informed the focus group discussions.^3^ Because no standardized tools captured the specific psychosocial effects of COVID‐19 relevant to this study, school belonging was examined relationally rather than as a psychometrically measured construct. It was indexed through survey‐based ecological determinants and correlates—including peer, teacher, and parental support; educational investment; intention to return; and emotional health—and through focus group narratives of connection, acceptance, recognition, refuge, and desire to return. Conducted across 2 years, this process reflects methodological plurality, with methods selected based on feasibility, cultural relevance, available resources, and their ability to capture both the population and phenomenon.

**TABLE 1:** Preparatory phases for the study: Creating culturally appropriate instruments.

Research design steps	Data type	Analytical process
Document analysis	COVID‐19 global reports on implications for adolescent girls	Thematic analysis using MAXQDA v. 2022.2
Coding	Publicly available interviews of adolescent girls in India done during COVID‐19	Thematic analysis using MAXQDA v.2022.2
Survey design	Creation of the COVID‐19 Gender Assessment Survey	Survey derived from thematic analyses
Focus Group Protocol	Survey 1 Results	Semi‐structured focus group protocol informed by document analyses and survey

The sequential mixed‐methods design was selected to align with the study's bioecological and intersectional research questions (Creswell & Clark, 2017; Greene, 2007). The survey provided descriptive and correlational evidence on conditions operating across ecological levels, including individual factors such as household labor, digital access, and emotional health; micro‐level relationships with parents, siblings, peers, and teachers; and macro‐ and chrono‐level conditions such as early marriage conversations, household financial stress, gender norms, and prolonged school closure. The focus groups then examined how girls interpreted these conditions, compared school and home environments, and experienced cross‐level interactions shaping school belonging.

### Positionality

The first author is a Hindi‐speaking Indian woman, a former rural schoolteacher and a international development professional with a focus on gender equity. She is currently an assistant professor of educational psychology in the U.S. She led the conceptualization, fieldwork, analysis, and writing. The second author, a Mexican American and first‐generation college graduate, is an assistant professor in educational psychology with expertise in belonging among culturally diverse populations. She contributed to data analysis and manuscript preparation. Both authors engaged in reflexivity throughout the research process to acknowledge how their lived experiences shaped data interpretation (Berger, 2015). Reflexivity was treated as an ongoing analytic practice rather than a one‐time positionality statement. Throughout survey design, focus group facilitation, transcription, translation, and coding, the first author maintained analytic memos documenting how her insider knowledge of gendered educational inequities in India shaped interpretation, while also noting moments where assumptions required closer examination. The two authors engaged in iterative analytic discussions to compare interpretations, question culturally familiar explanations, and ensure that themes reflected girls' accounts.

### Data collection

In 2021 and 2022, we administered surveys developed from the preparatory phase and, after analyzing the survey data, conducted focus groups across five KGBV schools; thus, quantitative and qualitative data collection overlapped during the study (Table 2). All study materials were reviewed by a field expert, local community members, and staff from SwaTaleem Foundation, a nonprofit working with KGBV communities, to ensure linguistic clarity, cultural appropriateness, and contextual relevance. This process strengthened content and face validity by ensuring that the instruments reflected participants' lived realities and locally salient concerns (Creswell & Clark, 2017); additional details regarding instrument development and validation are provided below.

**TABLE 2:** Surveys and focus groups: Comparison of the sense of school belonging and sociocultural challenges in different phases of the pandemic.

	Year 1, 2021	Year 2, 2022	Analysis
	One year into school closing	Two years into school closing	
COVID‐19 Gender Assessment Survey	*N* = 90	*N* = 60	Descriptive Statistics (mean, percentages), correlations, regression analysis, z score
Focus Group	*N* = 10	N = 10	Thematic Analysis, Constant comparative approach

SwaTaleem staff and KGBV schoolteachers also facilitated participant recruitment by identifying eligible girls and conducting outreach through school‐provided guardian phone numbers. Participants were drawn from the accessible pool of KGBV girls who could be reached during school closures, whose guardians consented, and who assented to participate.^4^ Data collection and analysis were interconnected: quantitative data identified broader patterns across participants, while qualitative data provided in‐depth insight into girls' experiences and interpretations. The two strands were integrated through narrative weaving, joint displays, and a unified discussion.

### Quantitative surveys

In 2021 and 2022, we administered the COVID‐19 Gender Assessment Survey to the participants which focused on exploring school belonging while identifying sociocultural challenges the girls faced.

#### Survey development

The COVID‐19 Gender Assessment Survey was developed from the preparatory phase analyses described above. We identified crisis‐relevant domains from the literature, document analysis, and publicly available accounts of rural adolescent girls during COVID‐19 and mapped these domains onto the study's bioecological framework. The resulting 39‐item survey assessed individual, relational, and sociocultural conditions relevant to girls' school belonging, including household labor, digital access, emotional well‐being, family decision‐making, peer and teacher support, early marriage conversations, and educational continuity (see Supplementary Material A). The first author translated the survey from English into Hindi.

As described in the Data Collection section, the translated instrument underwent community‐ and field‐based review for linguistic clarity, cultural appropriateness, and contextual relevance. A separate formal pre‐test with the target population was not conducted because of pandemic‐related access constraints and limited phone connectivity among KGBV girls. Because the survey consisted primarily of single‐item, crisis‐specific indicators rather than psychometric scales, construct validity was not assessed through factor analysis or internal consistency estimates. Instead, the instrument's content and face validity were strengthened through the preparatory development process and community review, and survey findings were subsequently triangulated with the focus group data.

#### Sampling and recruitment

Following ethics approval, school‐provided parent phone numbers were used to share study details via text and participants (aged 12–17) were given mobile recharges as incentives to support their online learning.

#### Data collection

The current study took place during the pandemic and used an online data collection method. See (Table B1) for the distribution of students by grade level. Notably, due to graduation, dropouts, or lack of phone access, the second‐year sample differed from the first.

#### Key variables

The COVID‐19 Gender Assessment Survey included seven sections to map the pandemic's impact on adolescent girls: *(a) Household Chores* included questions that helped learn how household chores affected school‐going girls differently than boys, *(b) Access to Phones* included questions to understand gendered digital access within homes during the pandemic, *(c) Social Support* included questions on the girls' social support systems at home during the school closures, *(d) Social Outcomes and Domestic Players* included questions on understanding the environment the girls faced at home and the main decision‐making players, *(e) Investment in School* included questions to understand the girls' motivation towards education and their plans to return to school, *(f) Emotional Health* 5 included questions that explored how the girls felt about their future, marriage prospects, and economic stress in the family as a result of the pandemic‐related school closure, and *(g) Demographics* included the participant's name, grade, number of brothers and sisters, and family size.

Designed as single‐item indicators and checklists to capture crisis‐specific ecological conditions that shaped girls' school belonging, the survey items included yes/no questions (e.g., whether there had been conversations about the participant's marriage), categorical frequency items (e.g., hours spent on household chores; amount of daily phone access), multiple‐choice items (e.g., who controls phone use; who provides academic or general support), and 1–5 sliding‐scale item on the extent of participation in specific household chores (See Supplementary Material A).^6^

#### Quantitative analysis

For 2021 (*N* = 90), we had 0.5% of missing data and for 2022 (*N* = 60), 2.5%; both years are within the acceptable range (Schafer, 1999). To assess the relationships between variables and compare key variables across groups, we used Pearson's correlation. For emotional health, we created a “difficult emotion” count variable by coding whether girls endorsed sadness, anxiety, anger, or hopelessness in response to the emotional health items. Endorsed emotions were coded as 1 and non‐endorsed emotions as 0, then summed to represent the number of difficult emotions reported. Early marriage conversations were operationalized as a binary indicator based on whether girls reported that there had been conversations about their marriage during the pandemic. This item captured the presence or absence of such conversations, not their frequency, intensity, or content; the qualitative focus groups provided additional detail on the meaning and context of these conversations.

To assess change across years, we conducted a two‐proportion z‐test comparing the percentage of girls who reported family conversations about their marriage in 2021 and 2022. We then conducted a linear regression to examine whether reported family conversations about early marriage were associated with girls' difficult emotion scores, controlling for household chores and access to technology. All analyses were done in R (Version 2024.04.2 + 764).

### Qualitative focus group with adolescent girls

The first focus group took place in January 2021 while the second focus group took place in January 2022. Both focus groups took place after the surveys.

#### Sampling and recruitment

Ten survey participants were randomly selected and invited to participate in the first online focus group via Zoom. Ten different girls were randomly selected for the 2022 focus group. Both focus groups were conducted in Hindi over Zoom and lasted 60–90 min.

Participants were drawn from the same KGBV schools and therefore shared a common institutional context, although they were not necessarily from the same grade. As a result, some girls may have known one another through school; however, the focus groups were not organized around pre‐existing friendship groups. The first author used a semi‐structured protocol and invited girls to respond conversationally as well as prompted quieter participants to ensure broad participation. Additionally, because the focus groups were conducted online during lockdown, the research team and nonprofit staff communicated with participants in advance about joining from as private a space as possible. However, because many girls lived in multigenerational households with limited private space, full auditory privacy could not be guaranteed. Participants were reminded that participation was voluntary, that they could skip any question, and that identifying information would be removed from transcripts.

#### Focus groups

We developed a semi‐structured focus group protocol (Supplementary Material C) informed by the document analyses and first survey results, as outlined in (Table 3). These discussions enabled us to delve deeper into the day‐to‐day experiences of the adolescent girls during the school closures and how their school belonging was impacted. For instance, under “structural determinants of gender inequalities,” we asked questions aimed at unpacking how household chores were assigned in accordance with gender roles and if the pandemic experience was different for people from different communities.

**TABLE 3:** Focus group semi‐structured protocol.

Category	Sample prompt
Experiences during COVID‐19	Can you walk me through your day at KGBV school versus your day in your home during the COVID‐19 lockdown?
Unpacking school belonging during COVID‐19	Did your feelings of belonging to the school and in general to education, change due to COVID‐19?
Structural determinants of gender inequalities	How have household chores disrupted your education? What role do the family members play in chores, etc.
Questions specific to Year 2: Changing life experiences	How have you been feeling after the second COVID‐19 wave? How have your plans changed?

Focus groups generated data through interaction, and we attended to how participants compared, affirmed, or extended one another's accounts. These interactions informed thematic development around household labor, peer connection, digital access, and school belonging. These were transcribed and translated into English by the first author.

#### Qualitative analysis

We coded the transcripts in MAXQDA version 2022.2 based on the bioecological model and employed a constant comparative approach (Glaser, 1965) when looking for similarities and differences across events, situations, and actors from the discussions at the two time points. Using the code matrix browser and profile comparison chart, we identified the distribution of codes and the intersection between codes across the focus groups (Table D1). We studied emerging macro and micro patterns through extensive data matrices in MAXQDA (Tracy, 2019).

To strengthen trustworthiness and reduce interpretive bias, we drew on validation strategies described by Creswell and Miller (2000). First, because the first author transcribed and translated the focus groups, translated transcripts and emergent interpretations were reviewed with the non‐profit staff familiar with the local linguistic, cultural, and educational context. Second, the authors engaged in analytic debriefing to compare interpretations and examine how researcher positionalities shaped coding and theme development.

### Mixed methods integration

Integration in this sequential mixed methods approach took place at several stages during the design, methods, analysis, and interpretation phases (Fetters et al., 2013). We initially analyzed quantitative and qualitative data separately according to the steps outlined above. The narrative weaving took place by integrating results from the two timepoints of the study for each of three levels of the bioecological model (individual, micro, and macro/chrono factors). In doing so, we evaluated how well the data integration aligned, determining whether there were confirmation/convergence, expansion, or discrepancies between the datasets (Fetters et al., 2013). For example, survey findings identified temporal shifts in household labor, phone access, sibling support, difficult emotions, and early marriage conversations, while focus group narratives explained how these shifts were experienced by girls as changes in routine, agency, relational support, and connection to school. The findings that emphasize how the integrative approach contributes to the overall interpretation drawn across the two datasets are presented together.

## RESULTS

We conceptualize school belonging as a multidimensional, ecological, and relational experience reflected in girls' connections with peers and teachers, recognition and being heard, perceived fit, opportunities for agency, and continued aspirational attachment to school (Table D1). Rather than relying on a single survey scale, we identify belonging across survey indicators and focus‐group narratives. Findings are organized across the bioecological model's three levels (Figure 1), showing how household labor, digital exclusion, teacher absence, early marriage discussions, and prolonged school absence weakened belonging, while memories of dormitory life, peer relationships, teacher recognition, and educational aspirations sustained it. The following sections examine each level and integrate survey (Table 1) and focus‐group (Table 4) findings across both time points.

**TABLE 4:** Survey results from the gender assessment survey.

Items in the survey	Year 1, 2021	Year 2, 2022
	*N* = 90	*N* = 60
Girls spoke to their brothers but never asked them to help with household chores	52%	71%
Girls whose brothers refused household chores due to gender roles over ability to so	21%	71%
Girls whose father/uncle/brother had a phone they borrowed during the pandemic	68%	69%
Men's access to phones in the household	79%	84%
Girls with access to a phone <60 mins/day	48%	51%
Girls with no connection to peers	26%	14%
Girls seeking support from parents	44%	29%
Girls seeking support from siblings	48%	64%
Girls whose family's discussed getting them married	2%	20%
Girls with a family member losing a job	24%	33%
Girls whose families reported financial crises	76%	76%
Outcomes girls were most afraid of during school closures		
Early marriage	5%	2%
Loss of schooling	81%	60%
Staying in an unsafe environment	14%	9%
Reasons to seek support during school closures		
Academic challenges	64%	83%
Physical health related challenges (fever, flu, etc.)	18%	8%
Mental health related challenges (anxiety, stress, etc.)	17%	0%
Future related challenges (going back to school, pursuing goals, etc.)	18%	8%
Average distribution of time in daily household chores during the pandemic		
House cleaning	22%	15%
Cooking	13%	22%
Dishwashing	17%	24%
Washing clothes	14%	12%
Buying groceries	11%	8%
Taking care of elders or younger siblings	23%	19%
Average time spent per day on household chores	23%	29%
Girls who did household chores in the morning	63%	73%

### Category I. Individual factors/demographic characteristics

This section examines how individual‐level disruptions to routine, study time, digital access, and emotional well‐being shaped girls' school belonging by weakening their sense of academic continuity, fit, and connection to school.

#### Household chores

According to the survey, girls spent 26% more time doing household chores in the second year of COVID‐19 than in the first. The average time spent conducting household chores was up to 7hrs by the second year, and more than 73% reported doing this work in the morning. It is important to note that in the non‐pandemic period, the morning time was spent in the schools, away from household chores. A girl in grade 9 shared, “I just kept sleeping initially. Gradually I was told by family enough of rest now, get to work. So, I started cooking food in the family.” Thus, as schools remained closed longer, household chores gradually became a constant part of the girls' lives and an integral part of their routines.

#### Online education and access to digital devices

Access to educational resources remained constrained across both years, with digital inequities intensified by gender and cultural norms. KGBV schools did not provide regular online instruction, and even when devices were available within households, girls' access was often mediated by family hierarchies. As one sixth‐grade student said in 2021, “When there were online classes [in my brother's school], my brother was bought a phone. They didn't buy me a phone; families don't buy phones for girls.” A seventh‐grade girl added, “A phone had come for me also, but my elder brother said no to my using it, so it was taken away.” Brothers were also described as having greater freedom and fewer household responsibilities. As one participant explained, “Even when they are home, our brothers take the phone from us, so their time goes like that. Boys do not have to do any chores at home, so our experiences were very different during COVID‐19.” Together, these accounts show that digital exclusion reflected both the absence of school‐provided infrastructure and gendered household hierarchies governing access to available technology.

#### Emotional health

In 2021, a seventh‐grade student expressed sadness about the school closure and shared, “I felt a little happiness and little sadness (on the day school closures were announced). It was very cold, so school closing made me happy. But I was also sad, because exams were canceled, and I had done very good preparation.”

The variable ‘difficult emotion’ in the survey showed a medium correlation with early marriage conversations that took place in the families of the girls. This aspect is discussed later in detail under macro level factors, but it is crucial to note the impact of emotional health on other critical areas of adolescent girls. By year two, the impact of school closures became more critical for the girls in the higher grades, such as tenth grade, because this is when they prepare for school boards, the standardized public examinations conducted at the end of key educational stages in India.

At the time of the second focus group discussion in January 2022, the schools had just reopened, and the girls were 2 weeks into the school term after having lost nearly 2 years of schooling. As they spoke about their emotional well‐being during the prolonged school closures, many reflected on this loss. A ninth grader shared her experience of coming back to school:

My practice to study or memorize answers is gone; my teachers would give us tasks in school to learn questions and answers and I could do that but then when the schools closed, I lost this habit, and I was no longer able to learn everything I read. It felt like a burden to be able to read and memorize. Now that we are studying again, it feels very difficult to grasp things in the classroom.

The girls valued schooling for the opportunities it provides. Many girls brought up a crucial component of the school design: the dormitories. Dormitories not only served as residential spaces, but by design, they move the girls away from the more rigid gender‐based roles of home. In 2021, an eighth‐grade girl shared:

We can focus more here and study more. When we go home, there is always work to do, and we have to leave our studies and then do household chores. Not only are studies good, but we also study because our friends study. So, by watching them do this work, we also join in studying. At home, if someone is talking, I would start listening to them and talking with them. In the dormitory, every evening we used to play quiz and trivia, which was very interesting. We learned a lot in the dormitory—we learned so many games; our friends were here.

Dormitories served both as an academic and social refuge for girls. It highlighted the contrast between home, where domestic responsibilities interrupted learning, and the school space, where peer influence, structured routines, and communal learning fostered belonging.

### Category II: Micro‐level factors

This section examines school belonging as a relational experience shaped through family, sibling, peer, and teacher relationships. While household relationships often constrained girls' educational agency and access, peers and teachers emerged as central sources of acceptance, recognition, emotional safety, and connection to school.

#### Siblings

Gendered divisions of household labor became increasingly visible across the 2 years. In 2021, more than half of the girls reported that they spoke with their brothers but never asked them to help with household chores; by 2022, 71% reported this pattern (Table 4). A seventh grader explained, “We work more than our brothers. *Bhabhi* [sister‐in‐law] also does it. I have never asked my brothers to work, my brother is older than me—I never speak to him, I fear him.” Reports that brothers refused to help with chores because of gender role expectations also increased from 21% in 2021 to 71% in 2022. Girls' accounts therefore suggest that sibling relationships became an important site through which household norms around gender, age, authority, and domestic responsibility were enacted as school closures continued.

#### Teachers

By the second year, teachers were starting to phase out of the girls' lives due to school closures and irregular communication channels. Reminiscing about the old days, one sixth grader shared:

In the morning, ma'am would give one chapter to revise in class. In the evening, we would tell her about the chapter. Everyone would go to their rooms; I would do it first and ma'am would send me to call other girls from their rooms.

Teachers had once been steady sources of care, warmth, and trust—figures who anchored the girls' school belonging. The missing role of teachers in the girls' lives was a constant reminder of the threat to school belonging and an indication that those relationships would have to be rebuilt once the schools reopened.

#### Peers

Girls continued to talk about their peers throughout the 2 years, identifying them as friends with whom they had fun, sought help, and studied. However, in the second year, their description of these peers started to change to a comparison of their feelings of acceptance and belonging when they thought of their peers compared to their relationship with parents at home. An eighth grader explained this succinctly:

We have our friends in school, we feel more belongingness and acceptance here among friends. At our homes, we are not able to share everything. Then on top of that, we don't know if others will make fun of what we say: this doesn't happen in school.

Friends often served as safe outlets for thoughts and emotions that girls may not share with others. These relationships offer care and affirmation, and trusted friendships can act as a buffer against feelings of exclusion.

### Category III: Macro‐ and chrono‐level factors

This section examines how broader sociocultural and temporal forces, including early marriage risk, gender norms, caste/class inequality, and prolonged school closure, shaped girls' school belonging by threatening their educational continuity while also revealing their continued attachment to school as a site of future possibility.

#### Early marriage impact on school belonging and the role of sociocultural norms

Child marriage and dropping out of school are two common social outcomes for KGBV girls (Table D1). In the first year, none of the girls brought up permanently dropping out of school. However, by the second year talks of early marriage entered their homes, affecting them and their siblings. One ninth grader shared, “Two of my elder sisters got married during this time—my mother was all alone, so I did all the work at home. I was in eighth. One sister was one year older than me.”

Marriage rates within communities were often influenced by financial circumstances, serving as a catalyst for decisions related to early or arranged marriages. In a 2022 focus group, a student explained:

When one girl gets married, the family thinks, ‘Let's also get one more girl married because the financial cost is less.’ So, there were more girls getting married because of this reason around us. During COVID‐19, the marriage ceremony was smaller (and therefore, less expensive), so families were trying to get more daughters married during this time.

Instances like this reflect the current state of marginalized girls in which they are still considered financial liabilities by their families. The survey data substantiated this pattern: the percentage of girls who reported that their families had discussed their marriage increased from 2% in 2021 to 20% in 2022. A two‐proportion z‐test indicated that this increase was statistically significant, z = −3.74, *p* < .05, suggesting that early marriage conversations became more prevalent as school closures continued. Job loss within the family also increased during this period, from 24% in 2021 to 33% in 2022. We also found that reported family conversations about early marriage were significantly associated with girls' difficult emotion scores, *R* ^2^ = .14, *F* (3, 42) = 3.45, *p* = .025, controlling for household chores and access to technology (Table 5). In other words, girls who reported early marriage conversations also reported more difficult emotions, including anger, anxiety, sadness, and hopelessness.

**TABLE 5:** Correlations of macro level factors with difficult emotions.

	Difficult emotions*
Early marriage conversations	0.44 [0.26, 0.59]
Financial crisis	0.20 [−0.09, 0.39]
Will to return to school	0.11 [−0.099, 0.31]
Connection to friends	−0.10 [−0.3, 0.11]

* Difficult emotions was a category that asked the girls if they experienced emotions like anger, hopelessness, anxiety, and sadness during the pandemic.

#### Intersectionality, school belonging, and sociocultural norms

Themes of intersectionality were abundant in the girls' descriptions of their pandemic experiences. As part of the macro‐level factor, intersectionality highlights how broader societal structures, such as gender, class, and other social identities, interact to create unique experiences of privilege or disadvantage for individuals and groups. One eighth grader clearly explained this in a 2022 focus group,

Our experiences are shaped by who we are. Our gender and class. Last year gave a first‐hand experience of how difficult the COVID time was. Poor and rich did not experience COVID the same way; everything had become so expensive that people who had money or were getting their salaries were okay, but poor people faced the maximum difficulty. Many of us were hungry. There was also that the internal migrants were returning home in large numbers.

Thus, the girls agreed that COVID‐19 affected all—it did not differentiate between caste, class, or gender—but, depending on their identity, people experienced it differently. This intersectional lens was not limited to their descriptions of COVID‐19 experiences; it was indicative of their notions about a world in which social hierarchies have a pronounced effect on the quality of life. One student added:

Let's say there is a man who is rich and a brahmin [higher caste], versus a woman who is a Dalit [lower caste] and poor. Because he was rich, he could afford many things. That would not be true for the woman. And for women, it is anyway difficult to live in this world.

Girls highlighted their understanding of the multidimensionality of social identities, which influence human experience. These social identities are interlocked and contribute to the prejudice and discrimination marginalized communities suffer by acting together.

#### Aspirational attachment to school and future possibility

The girls overwhelmingly reported missing the school residence and activities with peers and friends, which allowed them to feel like responsible students and exercise agency that was often constrained at home. During the second focus group in 2022, all participants said they wanted to return to school; most also wanted to attend college and believed they could do so. Although COVID‐19 left them several years behind academically, they continued to express ambition and optimism: “We want to take tough board exams, so we know where we stand in the world and how we prepare to compete in future.” When asked how COVID‐19 had affected her plans, one tenth grader shared, “If schools are closed this year also my board class will suffer. My plan was to take *Jamia* test [a college diploma test] and that would fail. But I have not given up; I still have that plan intact.” These aspirations persisted despite the challenges the girls navigated daily.

Their continued investment in school and future aspirations provides important evidence that school belonging persisted even when physical access to school was disrupted. Across both years, girls described school not only as a place of instruction but also as a space where they could imagine themselves as students, friends, and future collegegoers. Their desire to return, take board examinations, pursue college, and maintain future plans suggests that belonging was sustained through memory, aspiration, and imagined continuity. Thus, school belonging was not only relational, grounded in peers and teachers, but also aspirational: school remained a site of agency, recognition, and future possibility.

## DISCUSSION

School closure during COVID‐19 provided a unique opportunity to explore the complex relationship between historically marginalized rural girls' school belonging and the effect of sociocultural challenges. In doing so, the study advances methodological approaches to understanding school belonging by employing a sequential, culturally grounded mixed‐methods design. Combining two waves of gender‐focused quantitative survey and qualitative focus groups allowed for both breadth and depth in examining how belonging shifted across time and context. Importantly, the survey was co‐designed based on formative desk review and prior community interviews, embedding the study in girls' lived realities and not merely abstract constructs. This approach echoes calls by Creswell and Clark (2017) for mixed methods research that centers not only integration of data, but integration of participant worldviews and sociohistorical context. Importantly, the temporal layering—comparing data from two pandemic years—addresses critiques that much belonging research is cross‐sectional. By showing how the relational and institutional conditions supporting belonging were disrupted, the study contributes to methodological innovations in understanding belonging as a fluid, contested process rather than a fixed outcome.

This study amplifies the voices of a multiply marginalized group—rural, school‐going adolescent girls from minority communities in Haryana, India—who remain largely absent from dominant educational and psychological research (Henrich et al., 2010). Their experiences of gendered labor, digital exclusion, and early marriage risk offer a powerful counterpoint to the WEIRD‐centric focus of most school belonging literature. By documenting how these girls navigate disconnection and resistance across time, the study responds to calls for epistemic and demographic diversification in psychological science (Artiles, 2019; Bang & Vossoughi, 2016). It affirms that belonging is not a universal experience but one shaped by intersecting structures of power—and that underrepresented youth must be central to redefining the construct itself.

Additionally, the study's use of Bronfenbrenner's bioecological model as an organizing structure helped to scaffold a multilevel interpretation of how school belonging is disrupted and reconstituted during crisis. While ecological models have been widely applied in developmental psychology, their utility in qualitative‐global South contexts has been limited. This study demonstrates how the model can be adaptively applied to capture interactions between individual factors (emotional well‐being, digital access), micro‐systems (family, peer, and teacher relationships), and macro‐level pressures (patriarchal norms, early marriage risk) without assuming cultural uniformity in these layers (Tudge et al., 2009).

We found that sociocultural factors at individual, micro, and macro levels shaped the conditions through which school belonging was sustained or disrupted. Figure 2 synthesizes the integrated qualitative and quantitative findings into a conceptual model of school belonging during prolonged school closures. The contrast between constrained agency at home and greater agency at school heightened the salience of school as a site of belonging. Household chores increased, limiting girls' time for education, and access to digital devices was restricted by gendered norms, with brothers often prioritized over sisters. Contrary to this, at school, girls experienced greater agency and intellectual, experiential, cultural, and social freedom that increased their sense of belonging. Girls' school belonging tended to be further enhanced if they experienced diminished agency at home. Additional influences included their mental health and future aspirations, both of which were intertwined with their experiences of belonging.^7^ To further understand how school belonging is entrenched within the sociocultural norms surrounding the girls, we introduce three key themes: Intersecting Marginalizations: Belonging under Structural Constraint, Home as Hierarchy: Micro‐Level Gender Intensification in Crisis and School as Refuge: Reclaiming Belonging through Institutional Memory.

**FIGURE 2:**
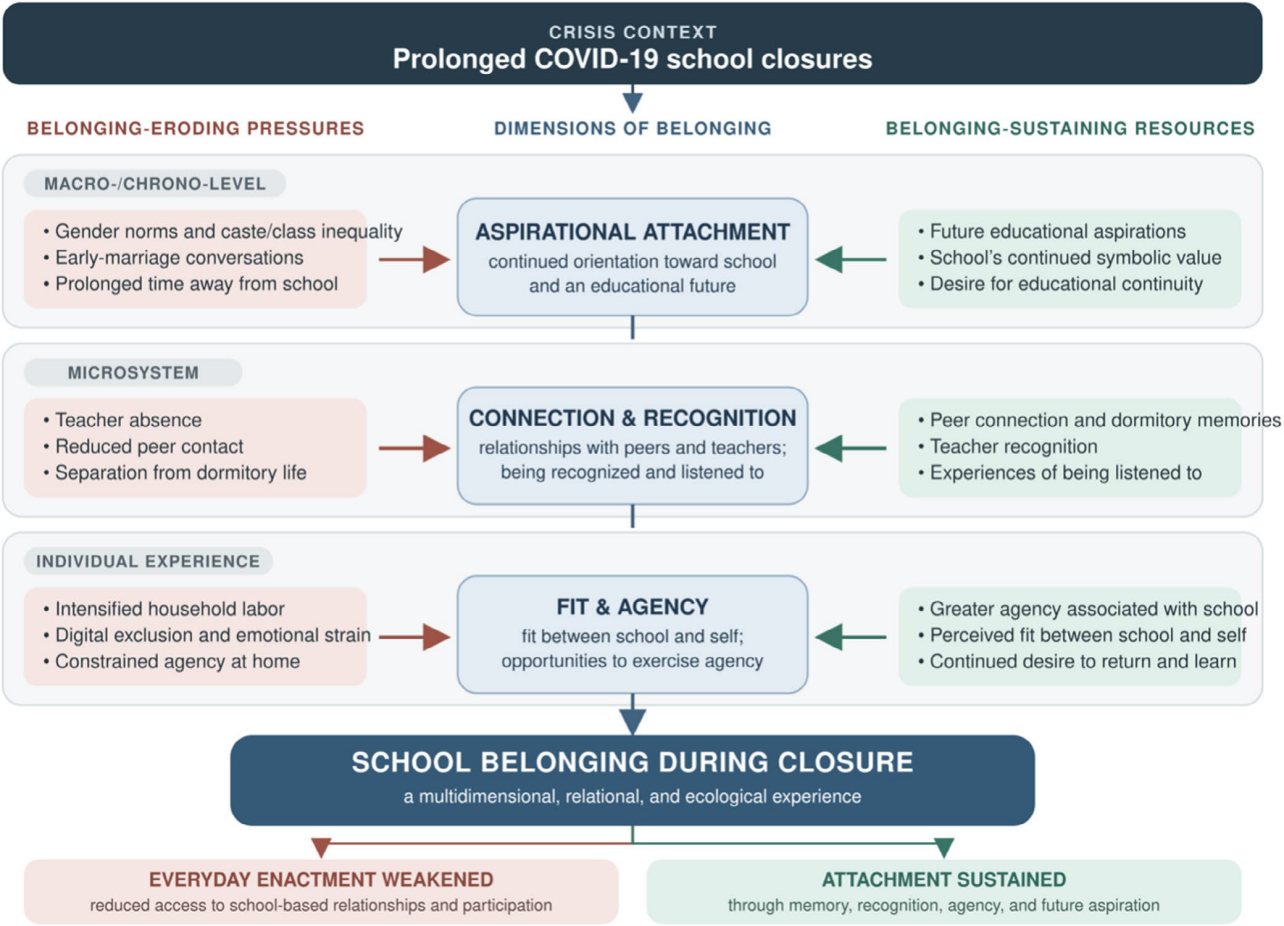
Bioecological Factors Shaping School Belonging During COVID‐19 School Closures.

### Intersecting marginalizations: Belonging under structural constraint

As girls shared their experiences and worldviews, intersectional processes emerged as a central explanatory lens for understanding variations in their school belonging during crisis‐related closures. Their narratives demonstrated that gender, caste, and class did not function merely as additive identity categories, but as structural systems of power that shaped their developmental trajectories, exposure to sociocultural constraints, and access to educational continuity (Cho et al., 2013; Collins & Bilge, 2020; Crenshaw, 1991; McCall, 2005). This reflects longstanding critiques in social psychology regarding the underutilization of intersectionality as an analytic approach for explaining patterned inequalities during public health crises (Bowleg, 2017). Accordingly, investigations of school belonging must account for these intersecting structures, particularly in contexts where multiple societal hierarchies are simultaneously activated and intensified (Tiwari, n.d.).

Bronfenbrenner's bioecological systems theory provides a useful scaffold for examining how these macro‐level social structures (e.g., caste hierarchies, gender norms, economic precarity) become instantiated in the everyday lives of girls through micro‐ and meso‐level interactions. The model's attention to nested systems helps illuminate how belonging is shaped by dynamic, non‐linear interactions among individual dispositions, family relations, and broader cultural norms (El Zaatari & Maalouf, 2022). Despite experiencing the same crisis context, girls' accounts showed that social hierarchies and differential access to resources fundamentally shaped their experiences. For example, girls from relatively wealthy families encountered fewer educational disruptions because financial stability enabled access to digital devices and reduced domestic labor, whereas girls from poorer households faced intensified chores, constrained mobility, and heightened emotional strain. Such disparities demonstrate that intersectionality is inherently structural—it explains why certain groups experience greater vulnerability and reduced belonging under crisis, not merely how many identities they hold (Anthias, 2013; Yuval‐Davis, 2006). In the Indian educational context, this structural lens is important because caste, class, and gender have historically shaped girls' access to schooling, recognition, and mobility (Tiwari, n.d.; Paik, 2014).

Importantly, gender norms operated across ecological levels to restrict girls' access to educational resources. This interplay of individual‐ and macro‐level factors in the bioecological model was mediated by actors such as microlevel family members. For instance, parents and siblings (micro level) were the gatekeepers to phones for the girls (individual level), thereby making digital access extremely gendered (macro level). These patterns illustrate how ecological and intersectional forces jointly structured the conditions under which belonging could be sustained, disrupted, or remade during a crisis.

### Home as hierarchy: Micro‐level gender intensification in crisis

Prior to crisis‐related school closures, KGBV girls spent substantial time in school—physically removed from the domestic sphere and the gendered labor expectations embedded within it. This removed components of household chores from the girls' lives and provided a set timetable to reinforce discipline, revision, and regular practice in the girls—all enhancing school engagement, which in turn contributes to school belonging (Korpershoek et al., 2020). Moreover, household chore allocation has long served as a mechanism through which families reinforce gender role socialization, with parents and elders assigning tasks in ways that delineate appropriate behaviors for girls and boys (McHale et al., 2003; White & Brinkerhoff, 1981). However, during the prolonged closures, the gendered organization of domestic space was not only reproduced but intensified, particularly through shifting sibling dynamics.

Unexpectedly, the findings revealed that brothers—often close in age—emerged as significant gatekeepers of girls' agency, particularly regarding access to educational technologies. Girls' accounts suggest that brothers often functioned as proximate actors through whom gendered household norms were enacted in relation to phone use, chores, and mobility. Because brothers were not interviewed, these findings should be read as evidence of how girls experienced sibling relationships within broader patriarchal household structures and not as claims about boys' motivations or internal beliefs. This dynamic simultaneously promoted boys' autonomy and reinforced girls' dependency, contributing to the re‐entrenchment of patriarchal norms at the micro‐ecological level. As Joseph (1994) argues, brother–sister relations often serve as conduits of patriarchal connectivity, transmitted through familial, cultural, and affective ties. Ridgeway and Correll (2004) further emphasize that such gender enactments are not merely internalized beliefs but are continuously reproduced through interactional hierarchies.

The intensification of gender roles was temporal: the longer the school closures persisted, the more deeply these patterns were normalized. The gradual erosion of institutional contact (e.g., with teachers or peers) left girls increasingly embedded in home‐based hierarchies where adolescent boys exercised growing influence. Although parental involvement at home plays a significant role in adolescent development and increases their school belonging regardless of how patriarchal the system is (El Zaatari & Maalouf, 2022), here we saw a well‐orchestrated ecosystem of family actors governed by traditional norms and, therefore, reproducing the same forms of discrimination that exist in society at large. While elder family members predictably shaped gender expectations, it was the adolescent brothers' rising authority that marked a significant shift—one that both reflected and amplified broader gender role divisions. This dynamic aligns with Hill and Lynch's (1983) notion of gender intensification, whereby adolescence becomes a period of heightened pressure to conform to culturally sanctioned gender roles. In this case, the crisis context accelerated and solidified gendered asymmetries, reshaping not only girls' roles in the household but also their psychological experience of autonomy, support, and school belonging.

### School as refuge: Reclaiming belonging through institutional memory

Schools operated not merely as sites of instruction but as affective and aspirational spaces that anchored adolescent girls' school belonging, even amid prolonged physical disconnection. Consistent with prior work (El Zaatari & Ibrahim, 2021; Longaretti, 2020; Porter et al., 2021), the school's ecological system supported both developmental and emotional needs, with peer and teacher interactions forming central relational processes in the construction of identity, community, and belonging. In fact, the experience of belonging was seen to be shaped by the reciprocal interaction between individual agency (person), educational environments (context), and socio‐temporal conditions (time) collectively—something that is seen previously (El Zaatari & Maalouf, 2022; Maurizi et al., 2013).

During crisis‐related school closures, the chrono‐system—the temporal layer of the bioecological model—became salient. As time away from school increased, girls' physical separation did not entirely sever their psychological and emotional ties to school. Rather, the memory of school as a space of routine, recognition, and relational security appeared to sustain a sense of institutional belonging. These findings echo Gallegos‐Castillo's (2006) concept of “suspended spaces”—mental or symbolic refuges through which marginalized youth temporarily distance themselves from oppressive social hierarchies. KGBV schools represented suspended spaces wherein girls could momentarily transcend gendered household roles and experience autonomy, collective care, and shared aspiration.

Importantly, the affective and symbolic salience of school was not limited to nostalgia. Girls articulated future‐oriented ambitions—such as passing board exams or attending college—which were tightly linked to their continued identification with school as a site of possibility. As hooks (1994) describes, education for structurally marginalized students can become “a practice of freedom,” enabling self‐determination and sociopolitical imagination. Even amid systemic constraints, the persistence of school‐based aspirations suggests that belonging was sustained through memory, desire, and the imagined continuity of educational opportunity. This is consistent with research emphasizing the transformative potential of school spaces for identity formation, especially for girls from underserved communities (Watson et al., 2002).

Ultimately, schools functioned not only as physical institutions but as relational infrastructures, offering access to peer networks, adult mentorship, and emotional safety—elements that underpin a durable sense of belonging. The erosion of these systems during the crisis magnified girls' vulnerability but did not erase their sense of connection. These findings reaffirm the need to understand school belonging not solely as an affective state bound by presence, but as a temporally sustained, socially constructed, and politically significant experience.

### Methodological lessons for research with underrepresented adolescents

Four methodological lessons follow from this design. First, cultural responsiveness should inform construct operationalization before data collection. We used contextual evidence to identify locally salient determinants, correlates, and expressions of belonging rather than treating an existing scale as culturally sufficient. Second, mixed‐method integration should connect research phases rather than occur after separate analyses: survey patterns determined areas for qualitative follow‐up, while focus‐group accounts identified the relational and structural processes underlying those patterns (Creswell & Clark, 2017; Fetters et al., 2013). Bioecological and intersectional frameworks further situated individual accounts within family, institutional, and structural systems. Third, access conditions are part of methodological validity. Remote data collection enabled research during school closures but underrepresented girls without reliable device access or auditory privacy. Fourth, culturally informed interpretation requires documented reflexivity. Analytic memos, review by locally knowledgeable staff, and cross‐author debriefing were used to examine translation and interpretive decisions (Berger, 2015; Creswell & Miller, 2000). Table 6 summarizes the study's methodological sequence, the culturally responsive purpose of each design decision, and corresponding recommendations for research with structurally marginalized adolescents.

**TABLE 6:** Methodological steps for culturally responsive sequential mixed‐methods research with marginalized adolescents.

Methodological stage	What this study did	Culturally responsive purpose	Recommendation for researchers
Contextual grounding	Synthesized relevant literature and analyzed published interviews with rural adolescent girls	Identified locally salient experiences that established measures might overlook	Begin with contextual evidence generated with or about the focal population
Survey development	Used the preparatory findings to develop contextually relevant survey questions	Reduced reliance on decontextualized measures	Explain how local evidence informed item content and wording
Local review	Used expert and community review to assess accessibility and cultural resonance	Improved linguistic and contextual appropriateness	Include locally knowledgeable reviewers and document resulting changes
Sequential connection	Used survey patterns to inform focus‐group questions	Allowed qualitative inquiry to explain quantitative patterns	Specify which quantitative findings shaped subsequent qualitative questions
Cross‐method integration	Interpreted survey and focus‐group evidence across two time points	Revealed both patterns and the relational processes underlying them	Integrate findings around shared constructs rather than reporting methods separately
Access and reflexivity	Examined remote participation, device access, translation, positionality, and missing voices	Made methodological power and exclusion visible	Report how data‐collection conditions affected participation and interpretation

## IMPLICATIONS

The findings show that school belonging is a dynamic, structurally mediated experience shaped by intersecting ecological and temporal factors. For adolescent girls in rural India, prolonged school closures did not merely interrupt education; they restructured the conditions of belonging at home, in school, and within girls' sense of self. These insights are especially consequential in South Asia, home to approximately 170 million adolescent girls—one‐third of the global total (UNICEF, 2023). Nearly half of global child marriages occur in the region, one in five girls is undernourished, and one‐third lack access to education, employment, or training opportunities (UNICEF, n.d.), making these structural conditions central to the global challenge of educational equity.

School belonging therefore functions not only as a psychological experience but also as a barometer of girls' broader structural conditions. Participants described school as a space of learning, aspiration, agency, and relational solidarity, and many retained a strong attachment to school despite prolonged physical disconnection. At the same time, school closures intensified gendered constraints at home. Notably, girls described adolescent brothers as household actors through whom patriarchal norms around domestic labor, mobility, and digital access were enacted. Educational interventions must therefore engage household dynamics, including parents and siblings, as part of efforts to promote gender equity, digital access, and educational continuity.

The findings also have implications for psychosocial support. Adolescents with stronger school belonging report lower emotional distress and greater well‐being (Arslan, 2018). During COVID‐19, girls who remained emotionally or relationally connected to school were better able to sustain aspirations, whereas those experiencing severe disconnection—often alongside technology denial or early marriage discussions—reported greater sadness, anxiety, and hopelessness. Consistent with earlier work (John et al., 2019), belonging may therefore function as a protective psychological factor. Gender‐sensitive mental health initiatives linked to schooling, including belonging circles, community storytelling, and group‐based resilience programs, may help rebuild this protective layer during post‐crisis recovery.

Methodologically, the study demonstrates the value of culturally grounded mixed methods approaches for capturing the complexity of belonging among diverse youth. Centering girls' cultural and linguistic contexts strengthened the validity and ethical grounding of the study and responds to calls for “epistemic inclusion,” in which the knowledge systems and interpretive frameworks of marginalized communities are centered rather than merely represented (Bang & Vossoughi, 2016).

From a policy perspective, these findings support calls for a gender‐transformative post‐crisis education agenda (UNESCO, UNICEF, & World Bank, 2021). Belonging should be treated both as a psychological outcome and as a systems‐level lever for countering forces that push girls out of school. This requires strengthening residential schools such as KGBVs, ensuring equitable digital access, preparing educators to recognize intersectional disadvantage, and incorporating belonging into education monitoring systems so that schools become environments in which girls feel seen, valued, and able to pursue their goals.

## LIMITATIONS AND DIRECTIONS FOR FUTURE RESEARCH

This study has several important limitations. First, COVID‐19 fundamentally shaped data collection. Because schools were physically closed, surveys and focus groups were conducted remotely via phone and Zoom, limiting opportunities for in‐person observation, consultation of school records, and consistent coordination with KGBV staff, who were themselves navigating crisis conditions. Remote data collection also restricted participation among girls without reliable phone or internet access, which should be considered when interpreting the depth and generalizability of the findings.

Second, sample attrition across the two study years may have introduced selection bias. The Year 2 sample differed from Year 1 because of graduations, school dropout, and loss of phone access. Girls who dropped out or became unreachable may have experienced the most severe disruptions to school belonging and the greatest risks of early marriage or permanent school exit. Their underrepresentation may therefore mean that the findings underestimate adverse outcomes and disproportionately reflect girls with more stable family and technological circumstances.

Relatedly, the digital divide was not simply a methodological constraint but part of the phenomenon under study. Preliminary data indicated that only 34% of KGBV girls had internet access at home, while phone access was shaped by gendered household norms that often prioritized brothers and fathers. Girls experiencing the greatest digital exclusion—and therefore potentially the sharpest disruptions in peer connection, teacher contact, and educational continuity—are likely underrepresented. Future research should deliberately include girls with varying levels of digital access and consider household‐based recruitment strategies to reach those disconnected from schools and digital networks. Although participants ranged across adolescence, the sample size and design also did not permit formal age‐ or grade‐based subgroup analyses; future studies should examine whether crisis‐related school closure shapes belonging differently across developmental stages.

Finally, the study was limited to 2 years of school closure. Longer‐term longitudinal research is needed to examine how girls reconstruct school belonging after reopening and whether educational aspirations are sustained over time. Comparative cross‐regional and cross‐national research could further illuminate how intersecting sociocultural and structural conditions shape belonging across contexts. More broadly, future research should examine school belonging during other crises, including climate‐related displacement, conflict, and migration (UNESCO, 2023), to better understand adolescent development under conditions of structural instability.

## AUTHOR CONTRIBUTIONS

**Ananya Tiwari:** Conceptualization; investigation; methodology; software; data curation; formal analysis; supervision; funding acquisition; project administration; writing – original draft. **Nidia Ruedas‐Gracia:** Writing – review and editing.

## FUNDING INFORMATION

The study was funded by the Margaret McNamara Education Grant, Washington DC, USA. It helped with the study design, analysis, and interpretation of results.

## CONFLICT OF INTEREST STATEMENT

The authors have no conflict of interest to disclose.

## ETHICS STATEMENT

All research was approved by the University of Illinois Institutional Review Board dated January 3, 2021 (Protocol #21518).

## PARTICIPANT CONSENT STATEMENT

Parental consent was secured through an opt‐in text message process, in which parents or guardians provided affirmative consent via text response. Youth assent was obtained digitally before completing the survey and participating in focus group discussions. Participation was voluntary, and adolescents could withdraw at any time.

## Supporting information


**Table B1.** Survey participants by grade levels.
**Table D1**. Code Overlap and Frequency Across Time: Bioecological Model Factors for School Belonging.

## Data Availability

The data that support the findings of this study are available on request from the corresponding author. The data are not publicly available due to privacy or ethical restrictions.

## Footnotes

^1^
Over 18% of girls in Haryana are married before the age of 18 (National Family Health Survey 2015, 2016), and more than half of adult women have less than 10 years of schooling (Chakravarty, 2018). In Mewat, where the average family size is twelve and fathers work as daily wage laborers or small‐scale drivers, educational continuity is precarious for many.

^2^
The KGBV system itself is also institutionally marginalized: 5000 schools like these are often excluded from national assessments, omitting at least 26% of the country's most deprived students who need to be represented in India's learning data the most (Goodnight & Bobde, 2018). Unlike better‐resourced public or private institutions, KGBV schools are rarely included in policy evaluations, donor reports, or academic studies. Consequently, their students—marginalized across multiple axes of caste, class, gender, religion, and geography—are effectively absent from the evidence base that informs education reform (Tiwari & Goodnight, 2024).

^3^
As part of the preparatory phase, we coded publicly available webinar minutes from Praxis's COVID‐19 Pandemic: Voices from Margins series, specifically Webinar 13, *Missing School: Adolescent Girls Marginalized by Growing Divide During Lockdown* (Praxis, 2020) (https://covid19voices.wordpress.com/wp‐content/uploads/2020/06/webinar‐13‐minutes.pdf). These materials included first‐person accounts from rural adolescent girls and youth advocates from Uttar Pradesh, Tamil Nadu, and West Bengal, as well as findings from a rapid Praxis study with adolescent girls across these states. These girls were not part of the present KGBV/Mewat sample; rather, the materials were used as a secondary, publicly available preparatory source to identify crisis‐relevant domains affecting marginalized girls' education during school closures in India. We coded these materials to identify recurring themes such as household labor, digital access, food and health insecurity, emotional distress, peer connection, early marriage risk, and desire to continue schooling, which informed the development of the COVID‐19 Gender Assessment Survey and focus group protocol.

^4^
Given the pandemic constraints and uneven phone access, we chose the process of recruitment through an accessible school‐community sampling frame rather than population‐level random sampling. In addition to supporting participant outreach, the non‐profit staff reviewed the survey items and focus group protocols for linguistic clarity, cultural appropriateness, and contextual relevance. This community‐based review provided an additional layer of content and face validity by ensuring that the instruments reflected locally meaningful concerns, including household labor, gendered digital access, family decision‐making, early marriage risk, and girls' desire to return to school.

^5^
For this question, girls could check more than one emotion. We created a difficult emotion variable that is a composite of the scores for sadness, anxiety, anger, and hopelessness as reported by the girls.

^6^
Because these items were not intended to form reflective latent scales, internal consistency estimates were not calculated. Instead, content validity was strengthened through the preparatory phase, translation into Hindi, and review by field experts and local community members.

^7^
Although the study was not designed or powered to formally compare age groups, there were developmental and school‐stage differences that emerged descriptively. Older girls, particularly those in grades 9 and 10, more often framed school closure in relation to board examinations, academic loss, future plans, and the risk of not returning to school, whereas younger girls tended to emphasize disruptions to daily routine, peer connection, and the immediate contrast between school and home. We therefore interpreted age‐related differences cautiously without reporting them as formal subgroup findings.
