# Supplementary material for: Belonging in crisis: A mixed‐methods exploration of school disruption among marginalized girls in India
Source: J Res Adolesc. 2026 Nov 14;36(4):e70327. doi: 10.1111/jora.70327 (PMC13768917; doi:10.1111/jora.70327)
Supplement: Supplementary file 1 [file JORA-36-0-s001.docx]

**SUPPLEMENTARY MATERIAL A**

**COVID-19 Gender Assessment Survey**

As part of Study Three, the COVID-19 Gender Assessment Survey was created to map the pandemic impact on adolescent girls in the Global South across six categories: household chores, access to phones, social support, social outcomes and domestic players, investment in school, and emotional health, in addition to demographics. All items were translated into Hindi.

| **I** | **Household Chores:** These questions are designed to know how the household chores have affected school-going girls differently than boys. It includes the types of chores girls mainly do at home, how much time they engage in them, and gender-related perceptions of household chores that got amplified during COVID-19. | |
| --- | --- | --- |
|  | **Questions** | **Response Choices** |
| 1 | Since school closure due to COVID-19, do you do more household chores (working in the kitchen, washing clothes, caring for others) than your brother(s)? | a. Yes  b. No |
| 2 | How much of these each household chores do you do? Indicate on the sliding scale (1–5). | a. Cooking b. Doing dishes c. Cleaning the house d. Washing clothes e. Buying groceries f. Talking care of elders or younger siblings |
| 3 | How much time do you spend doing the above things? | a. 1–3 hours b. 3–6 hours c. 6–9 hours d. 9–12 hours |
| 4 | Which part of the day do you do the most amount of household chores? | a. Morning b. Afternoon c. Evening d. I have to work the whole day. |
| 5 | If you have a brother, did you ask him to help you with the household chores anytime? | a. I am not supposed to talk to my brother. b. I talk to my brother but never asked him to help with household chores. c. I asked him but he refused. d. I asked him and he helps with some household chores. |
| 6 | What are the reasons given when you have asked for your brothers to share some household chores? Tick as many. | a. Household work is supposed to be done by girls only. b. Brothers or men do not have the ability to do household work. c. Elder brother should do some other kinds of work and younger ones are too young. To work d. Other ----- |
| **II.** | **Access to Phones:** These questions are framed to understand gendered digital access within homes during the pandemic. Questions include the type of phones households have, who has control over the phones as well as the feasibility of using them for digital education during COVID-19. | |
| **7** | What kind of phone do you have? | a. I have my own smartphone at home. b. My father/brother/uncle has a smartphone and I borrow it to use. c. My mother/aunt has a smartphone and I borrow it to use. d. I do not have a smartphone at home, I borrow it from outside the house. e. There is a simple button phone at home. |
| **8** | Who, mostly, gets to use the phone in your homes? | a. Father, uncles, brothers b. Mothers, aunts, sisters c. Myself |
| **9** | For how long you can use the phone every day? | a. Less than 30 mins b. Between 30 mins and 1 hour c. Between 1–2 hours d. Between 2–3 hours e. Between 3–5 hours f. More than 5 hours g. I do not get to use the phone at home. |
| **10** | If you are made to study via phone, what would be the best time for you? | a. Between 6–8 a.m. b. Between 8–10 a.m. c. Between 10 a.m.–12 p.m. d. Between 12–2 p.m. e. Between 2–4 p.m. f. Between 4–6 p.m. g. Between 6–8 p.m. h. I will not be able to study via phone. |
| **III.** | **Social Support:** These questions are intended to know the current social support systems that the girls have at their homes in the context of school closures during COVID-19. | |
| **11** | Do you have friends who live nearby? | a. Yes, there are friends from school but not classmates. b. Yes, I have classmates/friends nearby. c. No one from my school stays close to me. |
| **12** | How do you talk to your friends? | a. Through phone b. Through letters c. Through in-person meetings d. I don’t get to talk to my friends since the schools closed. |
| **13** | What is the main reason for which you had to seek support from someone else? | a. Academic challenges b. Physical health-related challenges (like headaches, fever, etc.) c. Mental health-related challenges (anxiety, stress, etc.) d. Relationship-related challenges e. Future-related challenges (being able to go back to school, pursue your goals, etc.) |
| **14** | Who has been helping you study after lockdown? | a. Teachers b. Parents c. Friends d. Siblings |
| **15** | After lockdown, who do you go for support in general? | a. Teachers b. Parents c. Friends d. Siblings |
| **IV.** | **Social Outcomes and Domestic Players:** These questions try to understand the kind of atmosphere the girls face at home and the main decision-making players. | |
| **16** | Who takes important decisions in your homes? | a. Father b. Uncle c. Brother d. Mother e. Sister f. Aunt |
| **17** | Staying at home after school closure, what are you most afraid of? | a. Marriage b. Staying behind in education c. Not being able to come back to school again d. Staying in an unsafe atmosphere e. Nothing |
| **18** | Did you come to know of any friend or school-going girl who got married during this time? | a. Yes b. No |
| **19** | Have there been conversations about your marriage during the COVID-19 pandemic? | a. Yes b. No |
| **20** | If yes, have you been asked for your opinion on the matter? | a. Yes b. No |
| **21** | Who in your family talks the most about your marriage? | a. Father b. Uncle c. Brother d. Mother e. Sister f. Aunt |
| **22** | Have there been money-related conversations for your marriage during the COVID-19 pandemic? | a. Yes b. No |
| **23** | Did anyone in your family lose a job in COVID-19? | a. Yes b. No |
| **24** | Some families reported having a financial crunch during COVID-19. Did your family also experience this? | a. Yes b. No |
| **V.** | **Investment in School:** These questions seek to understand girls’ motivation toward education and return to school. | |
| **25** | Are you able to study at home since the schools were closed? | a. Yes b. No |
| **26** | What are the challenges you face studying at home? | a. I don’t get time due to home chores. b. There is no phone at home. c. I have a phone but not a smartphone. d. I have a smartphone but no money for internet data. e. There is only one phone so either I don’t get it or get to use it very less. f. Other: ---------- |
| **27** | Do you feel like going back to school? | a. Yes b. No |
| **28** | What do you miss the most since schools shut down? | a. Friends b. School meals c. Teachers d. Studying |
| **29** | Do you think you will be able to come back to school? | a. Yes b. No, I like it at home. c. I want to but won’t be able to come due to family pressures. |
| **VI.** | **Emotional Health:** These questions seek to understand how the girls are feeling since the pandemic-related school shutdown with respect to their future, marriage prospects, and economic stress in the family. | |
| **30** | How have you been feeling generally since the school shut down due to COVID-19? | a. Sad b. Anxious c. Anger d. Hopeless e. Curious f. Excited g. No response |
| **31** | How did you feel when you heard someone in your family talk about marrying you? Tick as many. | a. Sad b. Anxious c. Anger d. Hopeless e. Curious f. Excited g. No response |
| **32** | How did you feel when you heard someone in your family lost a job? Tick as many. | a. Sad b. Anxious c. Anger d. Hopeless e. Curious f. Excited g. No response |
| **33** | How have you been feeling when you think about your future since the school was closed during COVID-19? Tick as many. | a. Sad b. Anxious c. Anger d. Hopeless e. Curious f. Excited g. No response |
| **VII.** | **Demographics** | |
| **34** | Name |  |
| **35** | Grade | a. 6th b. 7th c. 8th d. 9th |
| **36** | How many brothers do you have? |  |
| **37** | How many sisters do you have? |  |
| **38** | How many members are there in your house? |  |
| **39** | Do you get access to sanitary pads at home? | a. Yes b. No |

**SUPPLEMENTARY MATERIAL B**

**Table B1**

*Survey participants by grade levels*

|  | 2021 | 2022 |
| --- | --- | --- |
| Grades | N | N |
| 6 | 6 | 11 |
| 7 | 13 | 11 |
| 8 | 26 | 20 |
| 9 | 39 | 8 |
| 10 | 6 | 9 |
| Total participants | 90 | 59 |

**SUPPLEMENTARY MATERIAL C**

**COVID-19 and gender focus group discussions**

All focus group discussions took place in Hindi.

**Experiences during COVID-19**

- Can you share a little about the time when you heard that the schools were closing, and you had to go back home? How did you feel then?
- What did you do at home in the beginning?
- Can you walk me through your day at KGBV school versus your day in your home during the COVID-19 lockdown?
- Were there any challenges that you faced or are currently facing with education at home now that the schools are closed?
- How have you been feeling since the school closure?
- How have your plans changed because of COVID-19?
- Have there been any conversations around getting you or girls of similar age married? How did you feel when you heard something like this?
- Has someone in your family lost a job during COVID-19? How did that make you feel?
- Do you think you will come back to school? Why/Why not?

**Unpacking school belonging during COVID-19**

- How was home different from being at school? Do they seem like two different worlds?
- Did you get to participate with anyone or any group in some activities while staying at home?
- Do you feel connected to people in your new setting? Why/Why not?
- Did your feelings of belonging to the school and in general to education, change due to COVID-19?

**Structural determinants to gender inequality**

- How have household chores disrupted your education? (What role do the family members play in chores etc.?)
- Do you think the above experiences are different for boys or men? How?
- Do you think these experiences were different for people who may have had more money or were *Brahmin* (a prominent higher caste)?
- Are there any other challenges you wish to share?
- How are you coping with these challenges?

**Additional questions for focus group II**

- How did you feel when you realized that you would not go to school the second year in a row?
- How have you been feeling after the second COVID wave? How have your plans changed?

**SUPPLEMENTARY MATERIAL D**

**Table D1**

*Code Overlap and Frequency Across Time: Bioecological Model Factors for School Belonging*

| Code | Description | Quotation | Focus Group Code Frequency 2021 \| 2022 | |  |
| --- | --- | --- | --- | --- | --- |
| **Individual Factors: Demographic Characteristics** | | | | |  |
| COVID-19 Experience | Day-to-day events or occurrences that took place during the COVID-19 pandemic | I had my 2nd paper; I was going to school with my father. I saw girls coming back to their homes, so I asked them, why are you coming back? They told me school is closed today because Corona has come. I was very happy because there was no exam. I came back home; there I saw some masks. People [around me] said there is Corona, so be safe. I was very happy because there was no exam. (2021, grade 8) | 14 | 8 | |
| Household Chores | Work done within the household, e.g., cooking, and cleaning | They would go outside and travel around, we would work at home or watch TV; girls at least work a little, boys would just get to play around and spend time like that. (2021, grade 9) | 5 | 3 | |
| Access to Phones | Presence and access to digital devices, e.g., phones, during the school shutdown | My brother had a laptop, so sometimes I would get a chance to attend his online classes. (2022, grade 8) | 2 | 7 | |
| Coping | Girls’ behaviors directed toward effectively dealing with pandemic-induced difficult situations | First, I sat inside only, only give the object to the father. I didn’t come out (The girl sat in a kiosk shop and passed on grocery items to the father). But I knew how to keep an account of everything in the shop. Then when many a times my father was out—I took care of the shop—could do all accounting. (2021, grade 8) | 1 | 3 | |
| Emotional Health | The relative wellbeing of girls; how they felt and thought | I was sad because I could not meet friends after that. I couldn’t believe that this kind of disease had spread. (2021, grade 7) | 9 | 14 | |
| **Micro-Level Factors: Parent, Peer, and Teacher Support** | | | | | |
| Parent and Siblings | Interactions and experiences with household actors, specifically parents and siblings | We work more than our brothers. When I ask my brother, we end up fighting, sometimes I win though. (2021, grade 7) | 3 | 13 | |
| Teachers | The role of teachers; their presence and absence during school closure | I miss warden ma’am the most, I took her number and talk sometimes if possible. I had stayed with her in school dormitory that’s why I am really close to her. (2021, grade 8) | 5 | 0 | |
| Peers | The role of peers, their presence and absence during school closure | I am away from friends; I think about them a lot [repeats multiple times]. [We] used to have fun, help others, study with others . . . so much fun. We don’t have so much fun. (2021, grade 9) | 6 | 11 | |
| *School belonging | Feeling accepted, comfortable, and safe during the pandemic | Researcher: “Where do you feel a more sense of belonging? In school or at home?” Everyone said in chorus, “In school.” (2022) |  |  | |
| **Macro- and Chrono-Level Factors: Aspects of Culture and Context** | | | | | |
| Social Outcomes and Domestic Players | Societal and family pressures about early marriage, job loss in family | Two of my young sisters got married during this time—my mother was all alone, so I did all the work at home. I was in 8th. One sister had a college degree, and one was one year older than me. (2022, grade 9) | 5 | 14 | |
| Investment in School and Future Aspirations | Whether or not they should continue school as a result of the pandemic | I had thought this year I will give my best to education, and to school but I didn’t know that schools won’t open. I had thought that I would not miss even a single day at school and have 100% attendance. I had studied so hard before the school closed, and all that got wasted. (2021, grade 7) | 11 | 19 | |
| *Intersectionality | Social hierarchy themes, e.g., class and gender occurring together | Everyone saw COVID in the same light – irrespective of being rich or poor – but money was a real barrier in living through those times and conditions - so it was not the same for both  (2022, grade 9) |  |  | |
| Note: Code frequency indicates how many times participants mentioned a factor.  *Only first order code frequency directly emerging from the data, is reported. School belonging and intersectionality are secondary order codes formed by a combination of first level codes. For instance, intersectionality was coded under both “Social Outcomes and Domestic Players” and “COVID-19 experience”. | | | | | |
